# The role of gut microbiota in the regulation of standard metabolic rate in female *Periplaneta americana*

**DOI:** 10.7717/peerj.4717

**Published:** 2018-05-24

**Authors:** Paul A. Ayayee, Andrew Ondrejech, George Keeney, Agustí Muñoz-Garcia

**Affiliations:** 1 Department of Biological Sciences, Kent State University, Kent, OH, USA; 2 Department of Evolution, Ecology and Organismal Biology, Ohio State University, Columbus, OH, USA; 3 Department of Evolution, Ecology and Organismal Biology, Ohio State University at Mansfield, Mansfield, OH, USA

**Keywords:** Standard metabolic rate, Gut microbial assemblage, Nutritional stress, Antibiotic, *Periplaneta americana*

## Abstract

Insect gut microbiota contribute significantly to host nutritional ecology. Disrupting insect gut microbial assemblages impacts nutrient provisioning functions, and can potentially affect host standard metabolic rate (SMR), a measure of host energy balance. In this study, we evaluated the effect of disrupting gut microbial assemblages on the SMR of female *Periplaneta americana* cockroaches fed dog food (DF, high protein/carbohydrate (p/c) ratio), and cellulose-amended dog food (CADF, 30% dog food, 70% cellulose, low p/c ratio) diets, supplemented with none, low, or high antibiotic doses. Bacterial loads decreased significantly between diet types (*P* = 0.04) and across antibiotic doses (*P* = 0.04). There was a significant diet type x antibiotic dose interaction on SMR of females on both diets (*P* = 0.05) by the end of the seven-day experimental period. In CADF-fed females, SMR decreased linearly with decreasing bacterial load. However, SMR of DF-fed females on the low dose was significantly higher than those in the control and high dose groups. This is interpreted as a diet-dependent response by low dose DF-fed females to the loss of nutritional services provided by gut bacteria. Severe reductions in bacterial load at high doses reduced SMR of females on both diet types. This study provides insights into the potential role of gut bacteria as modulators of host energy expenditure under varying dietary conditions.

## Introduction

The standard metabolic rate (SMR), a proxy of energy expenditure at rest (Chown & Gaston, 1999), is a widely-quantified measure of an insect’s energetic state. The SMR reflects the balance between an insect’s energy usage (for growth and reproduction) and energy expenditure (foraging, eating, and digestion) (Nespolo, Lardies & Bozinovic, 2003). SMR is usually measured on resting, non-reproductive, and post-absorptive individuals (Schimpf, Matthews & White, 2012b). Insect SMR depends on a variety of factors, including the overall nutritional quality of intake diets (i.e., protein: carbohydrate: lipids balance), which dictates how much is consumed within a geometric nutritional framework (Raubenheimer & Simpson, 2003; Behmer, 2008) and how much energy is acquired following consumption and digestion (Chown & Gaston, 1999).

Nutrient intake regulation occurs through a variety of behavioral and physiological responses depending on the nutritional quality of diets. Insects feeding on nutritionally imbalanced diets can meet optimal species-specific protein/carbohydrate (p/c) ratios by compensatory feeding behaviors, such as increased foraging for food (if unrestricted) and or increased intake of low-quality food (if restricted) (Behmer, 2008). These behaviors are accompanied by metabolic and physiological changes that expend considerable amounts of host energy and have consequences for host SMR. However, these consequences are not easily predicted. Feeding on an imbalanced diet may induce metabolic suppression and lower SMR, or result in increased size of central organs that are metabolically more active, thus increasing SMR (Yang & Joern, 1994; Naya, Lardies & Bozinovic, 2007). For example, significantly lower SMR and fecundity were reported in harvestman, *Pachylus paessleri* (Opiliones) fed a carbohydrate-rich diet (carbohydrate 18.8%, protein 2.7%, lipids 0.1%, water 78%, total caloric content, 3.60 KJ/g) relative to a protein-rich diet (carbohydrate 1.1%, protein 21.2%, lipids 3.9%, water 72.7%, total caloric content, 5.2 KJ/g), an effect attributed to metabolic suppression (Naya, Lardies & Bozinovic, 2007). Suppression of SMR also occurs in other insects during periods of starvation or feeding on a poor quality diet, when insects rely on endogenous fat body reserves (Binner, Kloas & Hardewig, 2008; McCue et al., 2015). In contrast, locusts, *Locusta migratoria*, fed a low p/c (7:21) diet had significantly higher SMR than those fed a high p/c (21:7) (Zanotto et al., 1997). This suppression was attributed to elevated costs associated with increased physiological processing (enzyme secretion and digestion, and nutrient absorption) of higher amounts of low-quality food. Similarly, Yang & Joern (1994) observed an increase in gut size in grasshoppers fed diets with low protein concentrations compared with those fed diets with high protein concentrations. This increase in gut size might represent an increase in SMR since digestive organs are metabolically expensive. Finally, *Periplaneta americana* cockroaches have been reported to increase consumption of optimized artificial diets with high cellulose: dextrin ratios, relative to unmodified artificial diet and that increased consumption correlated with lower dextrin amounts (Bignell, 1978). Thus, the overall impacts of insect adaptations/responses to imbalanced diets on SMR are, therefore, not always linear to predict.

In addition to the effects on host physiology and compensatory behaviors, consumption of nutritionally imbalanced diets can also impact insect gut microbial functions, such as nitrogen provisioning (Douglas, 2009; Ayayee et al., 2014) and mediate insect hosts SMR. For example, *P. americana* cockroaches fed dog food diet had significantly higher bacterial load (cell counts) and higher amounts of microbe-derived metabolites (acetate and lactate) in their guts relative to those fed high-fiber diets (milled cereal leaves, corn cobs, or breakfast cereals) (Kane & Breznak, 1991; Gijzen et al., 1994). Microbe-derived acetate and lactate make up 14% of *P. americana’s* energy requirements (Kane & Breznak, 1991). These are essential intermediary metabolites needed for the biosynthesis of other metabolites, as well as crucial components of the Krebs’s cycle responsible for generating energy (Kane & Breznak, 1991). Thus, losing them represents a significant energetic cost to cockroaches feeding on high fiber diets. Although SMR data were not provided in these studies, diet-induced changes in the amounts of these metabolites are bound to impact cockroach SMR.

Finally, other factors, such as the presence of antimicrobials and allelochemicals in otherwise nutritionally balanced diets may render nutrients inaccessible to insects or metabolically expensive to extract (Douglas, 2009). This can impact host’s energy balance. Furthermore, antibiotics or allelochemicals in diets also disrupt crucial insect host-gut bacterial metabolic connections, as well as host-endosymbiont metabolic connections, compounding the negative effects on host’s energy balance. For example, the administration of the antimicrobial Metronidazole reduced gut bacterial loads, resulted in stunted growth, smaller hindguts, and thinner gastrointestinal in treated *P. americana* nymphs, relative to controls (Bracke, Cruden & Markovetz, 1978). This antibiotic also reduced body mass, decreased volatile fatty acid concentrations, and extended development times treated *P. americana* individuals relative to controls (Zurek & Keddie, 1996). In this instance, the observed effects were also associated with reduced endosymbiont concentrations in fat body and attributed to the loss of endosymbiont nutrient provisioning functions. Prolonged exposure of adult German cockroaches, *Blattella germanica,* to antibiotics also resulted in reduced numbers of the *Blattabacterium cuenoti* endosymbiont and shorter lifespans (Brooks & Richards, 1955; Richards & Brooks, 1958). Loss of gut microbial cellulase activity (Bignell, 1977; Gijzen et al., 1994) and supply of fermentative end products (Richards & Brooks, 1958; Cruden & Markovetz, 1987; Kane & Breznak, 1991), as well as some endosymbiont-associated functions (Brooks & Richards, 1955; Brooks, 1970; Zurek & Keddie, 1996) in treated versus control cockroaches, highlight physiological differences related following disruption of gut microbial associations. This is because clearing or depleting gut microbiota can interfere with bacterial nutritional services, forcing hosts to depend on the mobilization of internal reserves for energy production (Binner, Kloas & Hardewig, 2008; McCue et al., 2015) or compensate behaviorally and physiologically, which has implications for host SMR. Given the significant contributions of insect gut microbial assemblages to insect nutritional ecology and fitness (Douglas, 2009, 2013; Ayayee et al., 2014; Ayayee, Larsen & Sabree, 2016), it is likely that disturbing the gut microbiota would have an impact on insect host SMR, as is suggested for the role of gut microbes in energy regulation in vertebrates (Krajmalnik-Brown et al., 2012). However, studies that investigate the association between these two variables are lacking.

In this study, we investigated the simultaneous effects of diet and antibiotic on host SMR. We fed female American cockroaches, *P. americana,* diets with high or low p/c ratio, and exposed them to different antibiotic doses (none, low, and high). We measured SMR and initial body mass before the experimental period, as well as SMR, final body mass, change in body mass and gut bacterial loads after the experimental period. We did not anticipate any differences in SMR or body mass on day 1 because of the same initial rearing conditions. Given the reported compensatory feeding behaviors and lower bacterial loads in insects fed imbalanced diets, we predicted that females fed the control (no antibiotic added) low p/c diet would have higher SMR and lower bacterial loads than females fed the control (no antibiotic added) high p/c diet by day 7. We also predicted differences in the SMR of antibiotic-fed cockroaches, mediated by the combined impacts quality of diet (i.e., nutritional composition) and antibiotic dosage on gut bacterial loads, and the impact of dietary quality on compensatory feeding.

## Materials and Methods

### Insect rearing and selection diet and antibiotic preparation, and experimental design

*P. americana* females used in this study were obtained from the Department of Ecology, Evolution and Organismal Biology’s insectary, at The Ohio State University (Columbus, OH, USA). These insects were maintained in containers at ∼22 ± 2 °C in the insectary. Late instar female nymphs were collected from the insectary and kept together in a ventilated container at room temperature in the laboratory until final molt. Females were allowed to grow and feed for 1–5 days, but assigned an experimental group by day 5 (Fig. S1). Each female was placed individually into single plastic containers with the experimental diet. Water was provided daily by wetting cotton wicks placed in each container.

Virgin adult females were chosen for this study. The first 10–12 days, post final molt, represent an energy-intensive window in virgin females during which they mature sexually and allocate resources to oocyte development (Pipa, 1986). Virgin adult females more so than late-instar female nymphs and virgin adult males, undergo extensive physiological changes during this period before first oothecum deposition. Thus, resting, non-growing (adult), non-reproductive (virgin), and post-absorptive (starved) females (Schimpf, Matthews & White, 2012b) were chosen because dietary and antibiotic treatments were anticipated to have pronounced effects on their SMR during their maturation period.

### Experimental design

Chosen females were assigned to a dog food diet, DF (Red Flannel™ Hi-Protein Formula dog food, DF (PMI Nutrition, St. Louis, MO, USA), regularly used to maintain cockroach colonies, or a cellulose-amended DF, CADF, diet consisting of 30% dog food and 70% cellulose). Cellulose is often used in these dietary manipulations because it is not considered a phagostimulant nor a feeding deterrent (Bignell, 1978). It is also relatively stable and difficult to digest without gut microbial assistance. The types of microbial end products obtained from cellulose digestion by both bacteria and protozoa in CADF-fed cockroach gut are different from those in the DF-fed cockroaches (Kane & Breznak, 1991).

Low and high dose antibiotic diets were prepared by mixing with a solution of Chloramphenicol in water at concentrations of 0.025 mg/ml (∼0.03% of food weight and 0.25 mg/ml (∼0.3% of food weight), respectively. Chloramphenicol is a wide-spectrum bacteriostatic agent that inhibits bacterial protein synthesis (Dinos et al., 2016). The concentrations used in this study fall within the range of those used in previous studies (1–5 mg/ml) using *Plutella xylostella* (Lin et al., 2015), and the cockroach *B. germanica* (0.2% and 1%) (Brooks & Richards, 1955). Higher concentrations of Chloramphenicol than those used in this study, and for prolonged periods, have been shown to reduce both mycetocytes and the *B. cuenoti* endosymbionts in cockroaches (Brooks & Richards, 1955; Brooks, 1970). We do not anticipate this effect in our study. Control groups were fed DF or CADF diets mixed with water. Cockroaches were assigned to six experimental groups, (DF or CADF; control, low dose, and high antibiotic dose; *n* = 10 cockroaches in each experimental group). Incubating cockroaches for seven days on experimental diets was selected to minimize potential deleterious impacts of antibiotics.

### Measurement of SMR

Females were starved for 24 h before SMR measurements on day 1 and day 7 to ensure the animals were post-absorptive. Oxygen consumption was measured in a closed respirometry system, using the manual bolus integration method (Lighton, 2008). Body mass was measured before and after incubation. Females were individually incubated in air-tight glass syringes with 60 ml of dry atmospheric air for 60 min in an incubation chamber at 30 ± 2 °C. Reference air samples were collected in empty syringes filled with dry atmospheric air. There was a 5–7 °C temperature difference between room temperature and the incubation chamber. This temperature range falls within the ∼10 °C range above which SMR is expected to double by temperature (Harrison & Fewell, 1995; Dingha, Appel & Vogt, 2009; Streicher, Cox & Birchard, 2012). SMR measurements of insects at incubation temperatures that differ from laboratory or field conditions are not uncommon (Harrison & Fewell, 1995; Dingha, Appel & Vogt, 2009; DeVries, Kells & Appel, 2013). The 1 h incubation period might include discontinuous gas exchange cycles, as reported inaccounts for spiracle closures and openings in this and other cockroach species, which exhibit discontinuous gas exchange cycle, and is consistent with other similar cockroach SMR measures (Schimpf, Matthews & White, 2012a, 2012b). Following incubation, 40 ml of air was pulled from incubation syringes using a vacuum pump, at a flow rate of 260 mL/min (standard pressure and temperature (STP)) controlled by a mass-flow controller (Model 5850E; Brooks Instrument, Hatfield, PA, USA). The sample air was directed through a column of silica gel and ascarite to remove water vapor and CO_2_ respectively, then into an Oxzilla oxygen analyzer (Sable Systems International, Las Vegas, NV, USA). The percentage of O_2_ (VO_2_) in the sample air was then calculated and compared to the percentage of O_2_ in the reference air-tight syringe (20.95%) to estimate delta O_2_, which we used to calculate oxygen consumption using the following equation (Withers, 1977):

$${\rm{V}}{{\rm{O}}_2} = [{V_E}({{\rm{F}}_1}{{\rm{O}}_2} - {{\rm{F}}_{\rm E}}{{\rm{O}}_2})]/(1 - {{\rm{F}}_1}{{\rm{O}}_2})$$

where F_I_O_2_−F_E_O_2_ is the difference in the concentration of O_2_ between the reference air and the sample air (delta O_2_), and V_E_ is the flow rate of air (Withers, 1977). This value was then divided by time spent in the incubation chamber to determine oxygen consumption rate. Since we know the volume of air we sampled (40 mL), we could then calculate how much oxygen the animal consumed in 60 mL of the air-tight syringe. We did not take the volume of the animal, because it represents a small fraction of the total volume of air in which the animals were incubated. Our cockroaches are approximately 4 cm long, 1 cm wide and 0.7 cm tall (excluding the legs), and their shape can be approximated to that of an ellipsoid. With these values, the volume of a female will be on average around 1.5 cm^3^, or 1.5 mL, which corresponds to 2.5% of the volume of the syringe. Moreover, the size of the females was very similar, so we do not expect significant changes in the volume of each individual that can contribute to a significant bias in the calculation of oxygen consumption. Oxygen consumption was then converted into energy expenditure using 20.08 J/mL O_2_ (Schmidt-Nielsen, 1995)_._ To account for body mass in our estimates of energy expenditure, we calculated mass-specific SMR as SMR divided by body mass (mW/g). In addition to day 1 and day 7 SMR data, we also calculated the SMR ratio for each female as the quotient between SMR at day 7 and SMR at day 1. This ratio takes into accounts individual variability in responses across individual females before and during the experimentation period. A ratio of 1 indicates no difference in SMR, whereas a ratio lower or higher than 1 reflects a decrease or an increase in SMR over time, respectively.

### DNA extraction and quantitative polymerase chain reaction (qPCR)

On day 7, females were dissected, and the entire digestive tract removed. We measured 16S rRNA copy number of cockroaches using qPCR. We chose bacterial 16S copy number as our measured variable and not gut microbial community composition because we were interested in microbial function. Bacterial copy number is related to bacterial community succession (Shrestha, Noll & Liesack, 2007), as well as response rates of phylogenetically diverse bacteria to resource availability (i.e., function and ecological strategies) (Klappenbach, Dunbar & Schmidt, 2000). Thus bacterial 16S rRNA copy numbers serve as good proxies for microbial function, whereas community composition does not necessarily always relate easily to microbial function (Moya & Ferrer, 2018). Genomic DNA was extracted using the Qiagen DNeasy Blood and Tissue kit (Qiagen Inc., Valencia, CA, USA) as per manufacturer’s protocol following homogenization of gut tissue for 10 min. The bacterial primer pair 357F and 519R (1 μl each) (Turner et al., 1999) were used to amplify a roughly160 bp fragment in the V3 hypervariable region of the 16S rRNA gene (Chakravorty et al., 2007) in DNA samples via qPCR. The qPCR reaction mixes were comprised of 10 μl IQ™ SYBR Green Supermix (Bio-Rad Laboratories Inc., Hercules, CA, USA), 8 μl sterile milli-Q water, and 1 μl Gut DNA samples (∼16–18 ng/μl) to a final reaction volume of 20 μl. Plasmid standards for absolute quantification at eight different serial dilutions (10^8^–10^3^) in triplicate, as well as duplicates of template DNA samples, negative controls (milli-Q water) and positive controls (plasmid DNA), were used. Reaction conditions were an initial denaturation at 95 °C for 10 min, followed by 40 cycles of 95 °C for 15 s, 56.5 °C for 15 s, and 68 °C for 20 s, and a final elongation step of 62 °C for 20 s, followed by a melting curve step using the Realplex Mastercycler (Eppendorf, Westbury, NY, USA). Plasmid copy numbers of template samples were calculated based on the generated standard curve of plasmid serial dilutions after examination of melting curves, and bacterial load calculated as the number of plasmid copies per ng/μL.

### Statistical analysis

A mixed-model analysis was performed to determine the effects of diet and antibiotic dose on the measured variables (SMR, bacterial load, and body mass). Diet type (DF or CADF), antibiotic dose (control, low, and high), and their interaction were fixed factors, and individual female cockroaches, random factors. The student’s *t*-test was used to compare means among significant factors. Bacterial load data were log-transformed, but SMR and body mass data were not. Linear regression analyses were used to investigate the relationship between SMR and bacterial load, as well as body mass across fixed factors. All statistical analyses were carried with JMP 13 Pro (SAS Inc., Cary, NC, USA). We rejected the null hypothesis at *P* ≤ 0.05.

## Results

### Effects of diet and antibiotic doses on SMR

**Figure 1:**
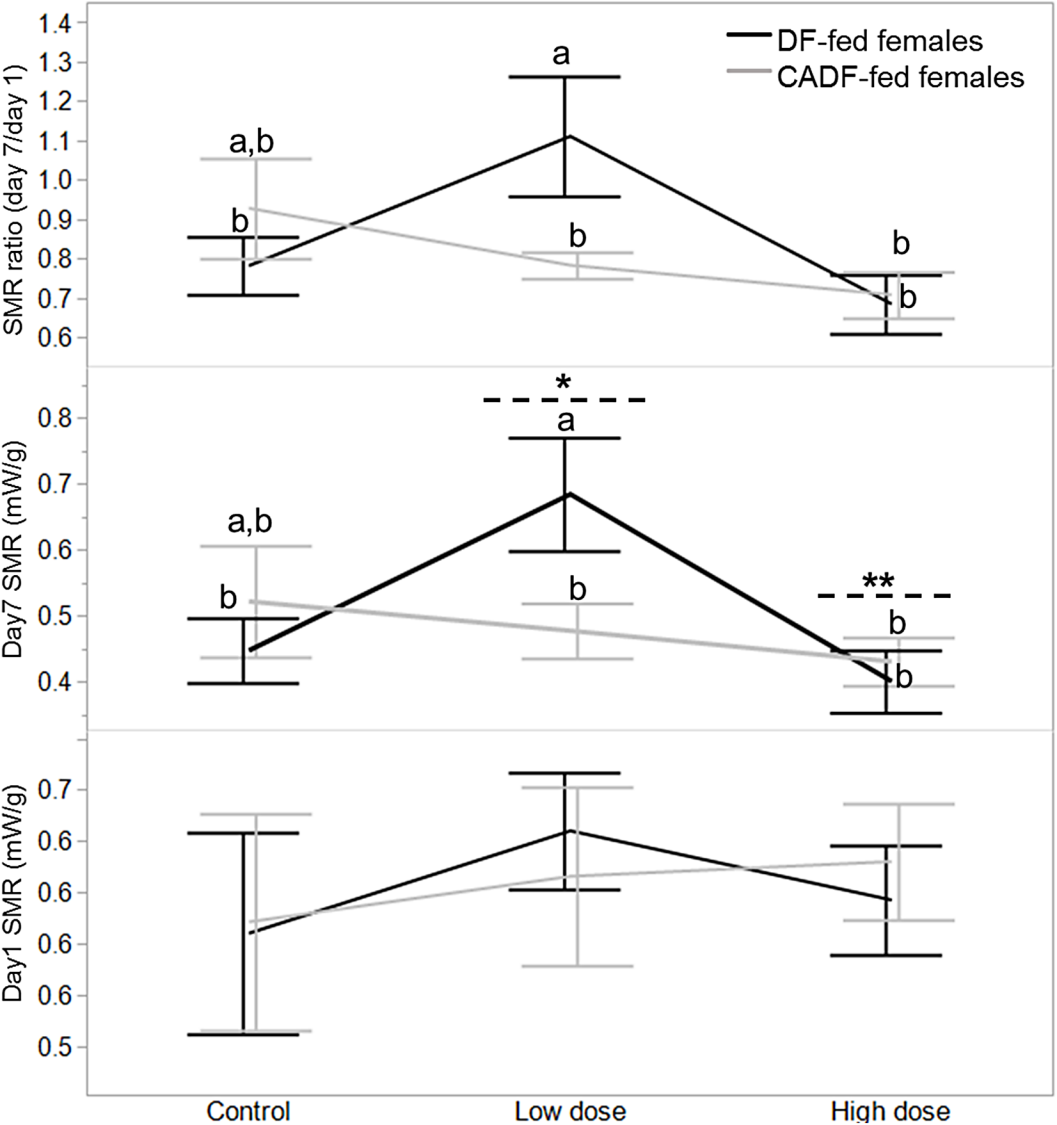
Effects of treatments on SMR. Day 1, day 7 and day 7/day1 standard metabolic rate (SMR) responses in DF-fed and CADF-fed *P. americana* females in control, low, and high dose antibiotic treatment groups. Significantly different diet x antibiotic interactions are indicated by letters, and significant differences among antibiotic doses are indicated by * and **. Bars represent standard errors of the means (S.E.).

On day 1, diet type (*F*_1, 46_ = 0.02, *P* = 0.88), antibiotic dose (*F*_2, 45_ = 0.34, *P* = 0.71), and their interaction (*F*_2, 45_ = 0.38, *P* = 0.68) had no significant impact on female SMR. SMRs were comparable for all females in each of the six categories (Fig. 1). On day 7, diet type (*F*_1, 46_ = 0.25, *P* = 0.62) had no significant impact of female SMR. However, antibiotic dose (*F*_2, 45_ = 3.13, *P* = 0.05) and diet x antibiotic interaction (*F*_2, 45_ = 3.20, *P* = 0.05) had significant impacts on female SMR. Mean female SMR (mW/g ± S.E.M) at the low (0.58 ± 0.05, mean ± S.E.) and high (0.42 ± 0.04) antibiotic doses were significantly different from each other, with mean control female SMR (0.48 ± 0.04) intermediate (Fig. 1). Across all six categories, SMR of DF-fed females at the low antibiotic dose (0.68 ± 0.06) was significantly different from the SMRs in the remaining four experimental groups except for control CADF-fed females (0.52 ± 0.06) (Fig. 1).

Diet (*F*_1, 46_ = 0.18, *P* = 0.67) and antibiotic dose (*F*_2, 45_ = 2.90, *P* = 0.08) had no significant impact on SMR ratio. However, the diet x antibiotic interaction increased in significance (*F*_5, 42_ = 3.64, *P* = 0.03) with SMR of DF-fed females at the low antibiotic dose (1.11 ± 0.09) similarly significantly different from SMRs from the remaining four experimental groups, except for control CADF-fed females (0.93 ± 0.09) (Fig. 1). Overall, both day 7 SMR and SMR ratio steadily decreased with antibiotic dose as anticipated by in CADF-fed females bay the end of the experimental period. However, in DF-fed females, SMR and SMR ratio were significantly highest at the low antibiotic dose than in controls but decreased abruptly at the high antibiotic dose (Fig. 1). Female SMR data in each of the six categories are shown in Table 1.

**Table 1:** Measured variables (SMR, body mass, and bacterial loads) of DF-fed and CADF-fed females across the control, low and high antibiotic dose treatment groups.

Diet type	Antibiotic dose	Day 1 SMR (mW/g)	Day 7 SMR (mW/g)	Day7/day 1 SMR	Log SMR ratio	Day 1 body mass (g)	Day 7 body mass (g)	Body mass difference	Bacterial load	Log bacterial load	Body mass ratio
Dog food	Control	0.75	0.57	0.77	−0.11	1.01	1.06	0.05	23,411	4.37	1.05
Dog food	Control	0.46	0.41	0.90	−0.05	1.04	1.13	0.09	3,868	3.59	1.09
Dog food	Control	0.51	0.40	0.78	−0.11	0.83	0.94	0.11	26,710	4.43	1.13
Dog food	Control	0.59	0.31	0.52	−0.28	1.17	1.36	0.19	35,861	4.55	1.16
Dog food	Control	0.59	0.55	0.94	−0.03	1.17	1.18	0.01	17,878	4.25	1.01
Dog food	High dose	0.53	0.40	0.75	−0.12	1.26	1.19	−0.07	24,148	4.38	0.94
Dog food	High dose	0.52	0.39	0.75	−0.12	1.16	1.19	0.03	34,969	4.54	1.03
Dog food	High dose	0.61	0.40	0.66	−0.18	1.06	1.15	0.09	11,776	4.07	1.08
Dog food	High dose	0.71	0.16	0.23	−0.64	1.24	1.26	0.02	10,266	4.01	1.02
Dog food	High dose	0.65	0.64	0.99	0.00	1.05	1.07	0.02	7,036	3.85	1.02
Dog food	High dose	0.64	0.46	0.73	−0.14	0.96	0.95	−0.01	4,211	3.62	0.99
Dog food	High dose	0.62	0.43	0.70	−0.15	1.16	1.15	−0.01	5,115	3.71	0.99
Dog food	High dose	0.49	0.33	0.67	−0.17	1.15	1.20	0.05	3,146	3.50	1.04
Dog food	Low dose	0.71	0.65	0.91	−0.04	1.11	1.28	0.17	25,422	4.41	1.15
Dog food	Low dose	0.56	0.65	1.15	0.06	1.10	1.07	−0.03	18,105	4.26	0.97
Dog food	Low dose	0.54	0.87	1.62	0.21	0.94	1.10	0.16	10,927	4.04	1.17
Dog food	Low dose	0.57	0.52	0.92	−0.04	1.10	1.24	0.14	14,561	4.16	1.13
Dog food	Low dose	0.64	0.69	1.08	0.03	1.03	1.06	0.03	12,119	4.08	1.03
Dog food	Low dose	0.62	1.25	2.01	0.30	1.21	1.32	0.11	20,654	4.32	1.09
Dog food	Low dose	0.56	0.57	1.03	0.01	1.19	1.17	−0.02	8,176	3.91	0.98
Dog food	Low dose	0.80	0.64	0.80	−0.10	1.31	1.30	−0.01	13,791	4.14	0.99
Dog food	Low dose	0.67	0.32	0.47	−0.33	1.29	1.21	−0.08	25,101	4.40	0.94
Cellulose-amended dog food	Control	0.74	0.39	0.52	−0.28	1.20	1.19	−0.01	36,523	4.56	0.99
Cellulose-amended dog food	Control	0.60	0.33	0.55	−0.26	1.08	1.11	0.03	5,706	3.76	1.03
Cellulose-amended dog food	Control	0.36	0.50	1.39	0.14	1.20	1.18	−0.02	7,533	3.88	0.98
Cellulose-amended dog food	Control	0.78	1.18	1.52	0.18	1.06	1.11	0.05	13,299	4.12	1.05
Cellulose-amended dog food	Control	0.77	0.46	0.60	−0.22	1.18	1.24	0.06	24,583	4.39	1.05
Cellulose-amended dog food	Control	0.40	0.50	1.24	0.09	1.00	1.10	0.10	19,949	4.30	1.10
Cellulose-amended dog food	Control	0.45	0.47	1.04	0.02	1.00	1.12	0.12	10,511	4.02	1.12
Cellulose-amended dog food	Control	0.55	0.40	0.73	−0.14	1.00	1.06	0.06	n/a	n/a	1.06
Cellulose-amended dog food	Control	0.62	0.47	0.76	−0.12	0.87	0.97	0.10	n/a	n/a	1.11
Cellulose-amended dog food	High dose	0.45	0.35	0.79	−0.10	0.82	0.87	0.05	8,683	3.94	1.06
Cellulose-amended dog food	High dose	0.64	0.26	0.41	−0.39	1.11	1.07	−0.04	n/a	n/a	0.96
Cellulose-amended dog food	High dose	0.67	0.41	0.61	−0.21	0.94	1.11	0.17	n/a	n/a	1.18
Cellulose-amended dog food	High dose	0.64	0.60	0.94	−0.03	1.02	1.22	0.20	5,036	3.70	1.20
Cellulose-amended dog food	High dose	0.63	0.41	0.65	−0.19	1.12	1.15	0.03	5,711	3.76	1.03
Cellulose-amended dog food	High dose	0.60	0.42	0.70	−0.15	0.99	1.18	0.19	5,345	3.73	1.19
Cellulose-amended dog food	High dose	0.57	0.50	0.88	−0.06	0.88	1.07	0.19	n/a	n/a	1.22
Cellulose-amended dog food	High dose	0.72	0.50	0.69	−0.16	1.09	1.15	0.06	n/a	n/a	1.06
Cellulose-amended dog food	Low dose	0.69	0.46	0.66	−0.18	1.11	1.12	0.01	18,774	4.27	1.01
Cellulose-amended dog food	Low dose	0.41	0.33	0.80	−0.10	1.31	1.31	0.00	n/a	n/a	1.00
Cellulose-amended dog food	Low dose	0.58	0.48	0.83	−0.08	1.32	1.34	0.02	n/a	n/a	1.02
Cellulose-amended dog food	Low dose	0.61	0.55	0.90	−0.05	0.88	0.92	0.04	17,351	4.24	1.05
Cellulose-amended dog food	Low dose	0.65	0.46	0.70	−0.15	1.28	1.37	0.09	3,450	3.54	1.07
Cellulose-amended dog food	Low dose	0.73	0.63	0.87	−0.06	0.97	1.16	0.19	10,296	4.01	1.20
Cellulose-amended dog food	Low dose	0.82	0.68	0.83	−0.08	0.94	1.09	0.15	1,492	3.17	1.16
Cellulose-amended dog food	Low dose	0.51	0.31	0.61	−0.21	0.98	1.03	0.05	n/a	n/a	1.05
Cellulose-amended dog food	Low dose	0.47	0.40	0.85	−0.07	0.95	1.06	0.11	10,359	4.02	1.12

### Effects of diet and antibiotic dose on bacterial load

**Figure 2:**
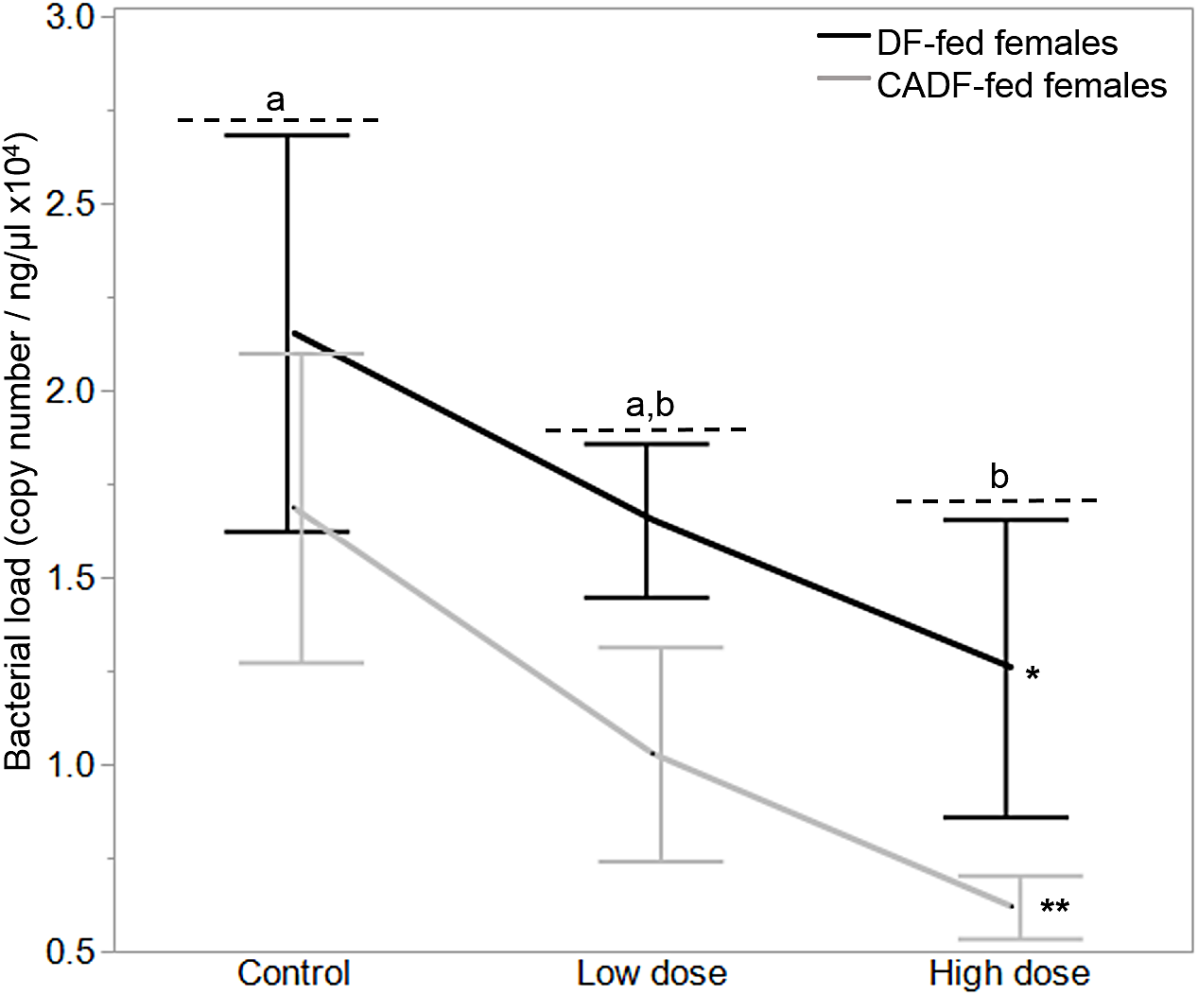
Effects of treatment on bacterial loads. Bacterial loads (16S rRNA plasmid copies per ng/μL) in DF-fed and CADF-fed *P. americana* females in control, low, and high dose antibiotic treatment groups after 7 days. Significant differences in bacterial loads among the three antibiotic treatments across both diet types (*P* = 0.04) are indicated by letters, and significant difference between diet types (*P* = 0.04) is indicated by * and **. There was no significant diet x antibiotic dose interaction. Bars represent standard errors of the means (S.E.).

On day 7, diet type (*F*_1, 37_ = 4.50, *P* = 0.04) and antibiotic dose (*F*_2, 36_ = 3.5, *P* = 0.04) significantly impacted female gut bacterial loads, but not their interaction (*F*_2, 36_ = 0.57, *P* = 0.57) (Fig. 2). Overall, on average, bacterial load (plasmid copies per ng/μL ± S.E.M was higher in DF-fed females (1.68 × 10^4^ ± 0.20) relative to CADF-fed females (1.11 × 10^4^ ± 0.22). Among antibiotic doses, bacterial loads (plasmid copies per ng/μL ± S.E.M) were on average significantly highest in control groups (1.91 × 10^4^ ± 0.26), followed by the low (1.34 × 10^4^ ± 0.24) and high (0.94 × 10^4^ ± 0.27) groups. Female bacterial load data in each of the six categories are shown in Table 1.

### Effects of diet and antibiotic dose on body mass

A mixed-model analyses of initial (day 1) and final (day 7) body masses (g), as well as change in body mass (g) (final–initial) were carried out to investigate the impacts of treatments on insect body mass, and how this explains observed effects on SMR. On day 1, diet (*F*_1, 46_ = 1.86, *P* = 0.18; DF-fed females = 1.10 ± 0.02, g ± S.E.M; CADF-fed females = 1.05 ± 0.02), antibiotic dose (*F*_2, 45_ = 0.83, *P* = 0.44; control females = 1.05 ± 0.03 g ± S.E.M; low dose females = 1.11 ± 0.03, high dose females = 1.06 ± 0.03), and their interaction (*F*_2, 45_ = 0.80, *P* = 0.46) did not vary significantly, as anticipated. On day 7, diet (*F*_1, 46_ = 0.86, *P* = 0.36; DF-fed females = 1.16 ± 0.02 g ± S.E.M, CADF-fed females = 1.13 ± 0.02), antibiotic dose (*F*_2, 45_ = 0.92, *P* = 0.40; control females = 1.13 ± 0.03 g ± S.E.M, low dose females = 1.20 ± 0.03, high dose females = 1.12 ± 0.03), and their interaction (*F*_2, 45_ = 0.10, *P* = 0.90) did not have any effects on body mass either. However, on day 7 diet x antibiotic dose interaction significantly impacted change in body mass over the seven-day treatment period (*F*_2, 45_ = 4.54, *P* = 0.018), whereas diet (*F*_2, 45_ = 2.76, *P* = 0.10; DF-fed females = 0.05 ± 0.02 g ± S.E.M, CADF-fed females = 0.08 ± 0.02) and antibiotic dose (*F*_2, 45_ = 0.56, *P* = 0.57; control females = 0.08 ± 0.02, g ± S.E.M; low dose females = 0.05 ± 0.02; high dose females = 0.07 ± 0.03) did not.

**Figure 3:**
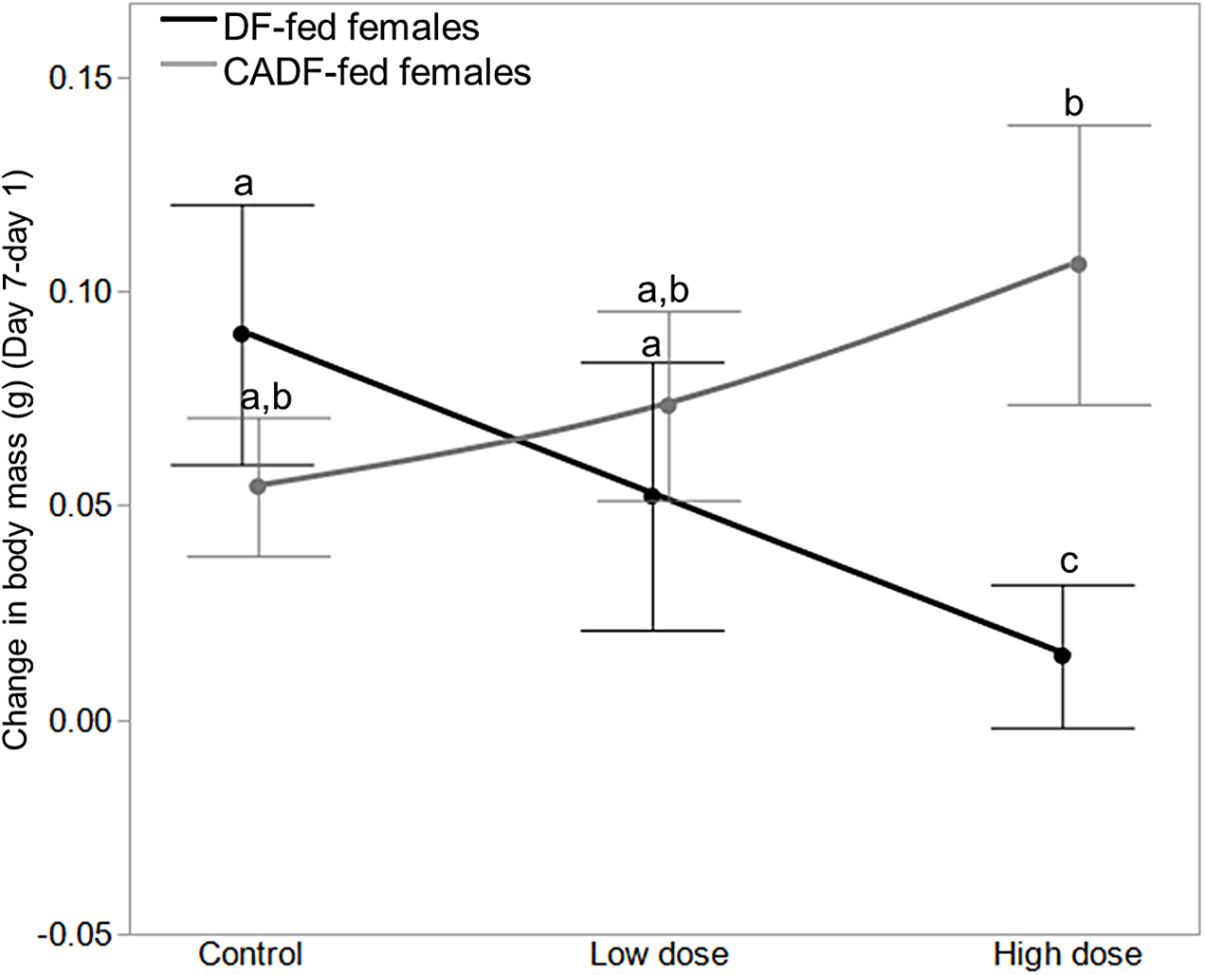
Effects of treatments on body mass. Change in body mass in DF-fed and CADF-fed *P. americana* females in control, low, and high dose antibiotic treatment groups. Significantly different diet x antibiotic interactions are indicated by letters. Bars represent standard errors of the means (S.E.).

Among the six treatment groups, change in body mass was significantly smaller in high dose DF-fed females (0.02 ± 0.02, g ± S.E.M) relative to control DF-fed females (0.10 ± 0.03), meaning control DF-fed females gained approximately 0.08 ± 0.03 (g ± S.E.M) more weight than high dose DF-fed females (Fig. 3). There were no significant differences among control, low dose, and high dose CADF-fed females, although changes in body mass increased and were highest in high dose CADF-fed females (0.12 ± 0.02, g ± S.E.M) (Fig. 3). However, there were significant differences in changes in body mass between high dose CADF-fed females (0.12 ± 0.02, g ± S.E.M) and low dose DF-fed females (0.04 ± 0.02, g ± S.E.M), and between high dose CADF-fed females and high dose DF-fed females (0.02 ± 0.02, g ± S.E.M) (Fig. 3). Thus, high dose CADF-fed females gained approximately 0.074 ± 0.03 and 0.091 ± 0.03 (g ± S.E.M) more weight, respectively, relative to low dose and high dose DF-fed females. An analysis of the day 7/day 1 mass ratios yielded the same trends and significant differences (data not shown). Mass data for females in each of the six categories are shown in Table 1.

### Relationships between metabolic rate ratio and bacterial load

**Figure 4:**
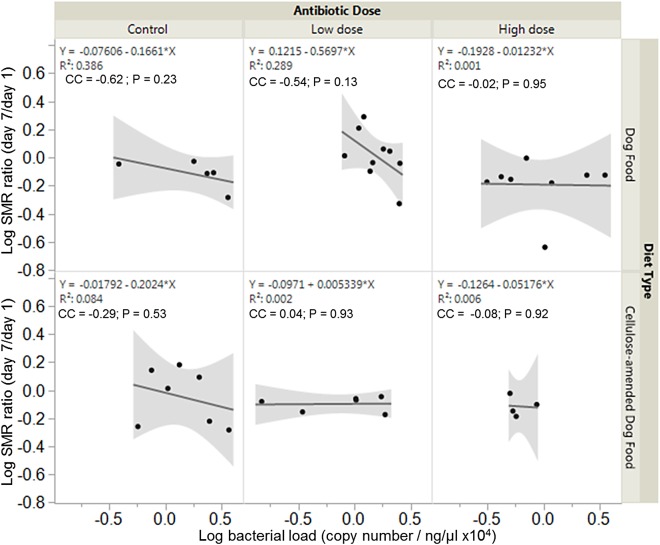
Relationships between SMR and bacterial load. Overall correlations between SMR and gut bacterial load in DF-fed and CADF-fed *P. americana* females at the three antibiotic doses. At control and high antibiotic doses, SMR of both DF-fed and CADF-fed females increased with decreases in bacterial load. At the low antibiotic dose, SMR of both DF-fed females increased with decreases in bacterial load but increased with increasing bacterial load in CADF-fed females.

Overall, there were no significant associations between bacterial load and SMR ratio across experimental groups. However, SMR ratio and bacterial load were negatively correlated in control DF-fed (correlation coefficient, cc = −0.62, *P* = 0.23) and CADF-fed (cc = −0.29, *P* = 0.53) females (Fig. 4). In the low antibiotic dose category, SMR ratio correlated negatively with bacterial load in DF-fed females (cc = −0.54, *P* = 0.13), but positively in CADF-fed (cc = 0.04, *P* = 0.93) females (Fig. 4). At the high antibiotic dose, SMR ratio correlated negatively with bacterial loads in both DF-fed (cc = −0.02, *P* = 095) and CADF-fed (cc = −0.08, *P* = 0.92) females.

### Relationships between metabolic rate ratio and change in body mass

**Figure 5:**
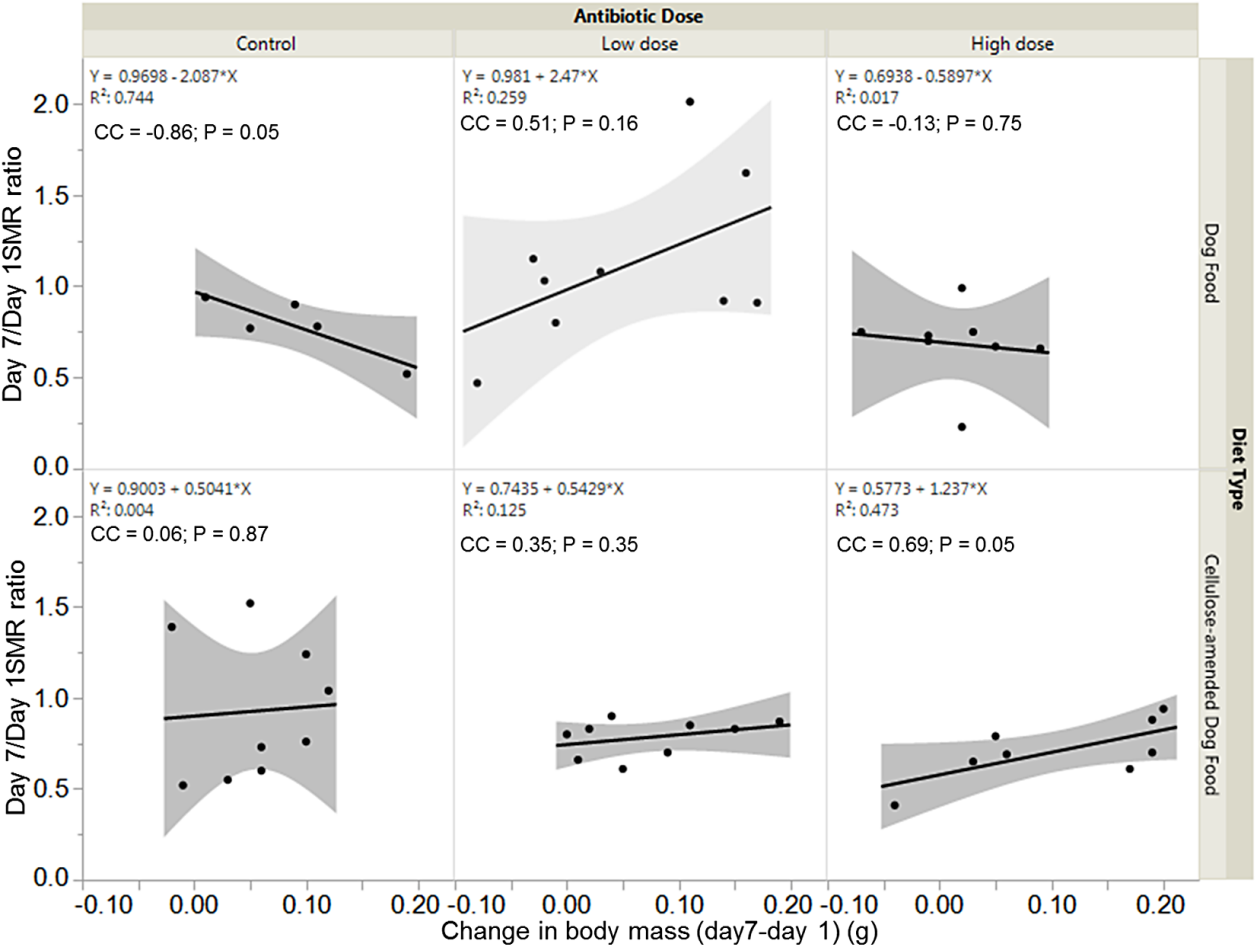
Relationships between SMR and change in body mass. Overall correlations between SMR and change in body mass in DF-fed and CADF-fed *P. americana* females at the three antibiotic doses. In CADF-fed females, SMR increased with increase in change in body mass across all three antibiotic treatment groups. In Df-fed females, SMR decreased with increases in body mass at both the control and high antibiotic dose but increased with increase in body mass at the low dose.

There was a significant negative association between SMR ratio and change in body mass for control DF-fed females (correlation coefficient, cc = −0.86; *P* = 0.05), but a significant positive association between SMR ratio and change in body mass for high dose CADF-fed females (cc = 0.69; *P* = 0.05) (Fig. 5). The relationships between SMR ratio and change in body mass were positive but not statistically significant for both control (cc = 0.06, *P* = 0.87) and low dose (cc = 0.35, *P* = 0.35) CADF-fed females. For DF-fed females, SMR correlated positively with change in body mass at the low dose (cc = 0.51, *P* = 0.16) and negatively at the high dose (cc = −0.13, *P* = 0.75), but were not statistically significant (Fig. 5).

## Discussion

In this study, the SMRs of virgin adult female *P. americana* cockroaches on differentially manipulated artificial diets are proposed to be mediated by gut bacteria. On day 1, none of the initial measured variables were significantly different among treatments. The lack of significant effects on day one were anticipated since all individuals at this point had been the protein-rich DF diet, with no antibiotics. By the end of the experimental period, a diet x antibiotic interaction significantly impacted cockroach metabolic responses SMR (*P* = 0.03), and diet type (*P* = 0.04) and antibiotic dose (*P* = 0.04) significantly impacted gut bacterial loads. The significant impacts of diet and antibiotic dosage on bacterial load, coupled with the significant diet x antibiotic dose interaction on SMR is interpreted as positive indications of gut bacteria-mediated contributions to host insects’ energetic state.

Although they were not statistically significant, the anticipated higher mean SMR ratio of control (no antibiotic) CADF-fed females (*N* = 9) (0.93 ± 0.09) relative to control DF-fed females (*N* = 5) (0.76 ± 0.13) was observed. The medium Cohen’s effect size value (*d* = 0.50) between these groups suggests a moderate practical or biological value of this difference, with approximately 69% of the CADF-fed females above the mean for the DF-fed group (Cohen, 1977). The current explanation for this would be that females engaged in compensatory feeding behaviors on the low-quality diets (Raubenheimer & Simpson, 2003; Behmer, 2008), and the associated metabolic costs of increased ingestion, digestion, and absorption (Zanotto et al., 1997), accounted for the observed higher SMR. Increases in body and gut sizes (Yang & Joern, 1994) on low-quality diets are often used to corroborate compensatory feeding behaviors and the higher SMR, although this relationship is not always linear. In this study, an increase in SMR in control CADF-fed females was not accompanied by a significant increase in body mass nor change in body mass relative to control DF-fed females, as might be expected by day 7 (Fig. 3). This may be attributed to the duration of the experimental period or might be indicative of an additional contributing factor. For example, the observed higher SMR of control CADF-fed females might be explained by the impact of low-quality diet, such as the CADF diet, on reducing insect gut bacterial loads (Kane & Breznak, 1991). Average bacterial load in DF-fed females across all antibiotic treatments was significantly higher than CADF-fed females. Furthermore, there was a ∼1.27 magnitude difference in bacterial loads between control DF-fed females and CADF-fed females (Fig. 2). Reductions in gut bacteria load may consequently be followed by decreases in the amounts of microbe-derived metabolites, such as lactate, acetate, and butyrate (Kane & Breznak, 1991; Gijzen et al., 1994), which are intermediary metabolites crucial for generating energy (Kane & Breznak, 1991). Furthermore, diet-induced decreases in lactic acid producing bacteria (*Lactobacillus, Streptococcus*, and *Enterococcus*) abundances were determined in cockroaches fed diets besides the dog chow (Kane & Breznak, 1991). Although community composition was not examined, control DF-fed females may be expected to similarly have higher abundances of lactic acid producing bacteria genera relative to control CADF-fed females. Thus, diet-induced reductions in gut bacterial loads and disruption of host-gut bacteria metabolic connections may be connected to increased compensatory responses leading to higher SMR in control CADF-fed females relative to control DF-fed females. Although not statistically significant, potential diet-dependent impacts of gut bacteria in mediating SMR can be inferred from the negative associations between SMR ratio and bacterial loads for both control DF-fed (*P* = 0.23) and CADF-fed (*P* = 0.53) females, with higher bacterial loads resulting in lower insect SMR ratios. Potential diet-dependent impacts of reduced bacterial loads (and associated microbial functions) on host physiology can be inferred from the negative correlation between SMR ratio and change in body mass in control DF-fed females (*P* = 0.05; bigger individuals have lower SMR). Potential diet-dependent impacts of reduced bacterial loads on host physiology can be inferred from the positive association between SMR ratio and change in body mass in control CADF-fed females (*P* = 0.87; larger individuals have higher SMR). Lack of a statistical significance of differences in SMR ratio between control DF-fed and CADF-fed females in this study may be the result of the short experimental period or number of insect replicates. However, results are promising and indicative of gut bacteria-mediated effects on insect responses to dietary quality. Long-term studies with higher replicates investigating gut bacteria-mediated effects on host SMR on similar DF and CADF diets are ongoing.

The importance of gut bacteria in mediating diet-dependent SMR responses is further reflected in SMR responses of both DF and CADF diets at the low and high antibiotic doses. Disruption of host-gut bacteria metabolic connections through dietary manipulations (nutritional composition and presence of antibiotics) may be underscoring host SMR responses in ways that remain to be clearly elucidated. The high SMR of low dose DF-fed females may be attributed to longer food retention in the digestive tract for extended nutrient extraction by both host and microbial enzymes and increased reliance and utilization of stored food reserves. Both of these may be in response to the presence of antibiotic in the DF diet which lowers overall quality of the diet despite the high p/c ratio, as well as reduced bacterial load and the loss of microbial nutritional services that implies. The absence of compensatory feeding in low dose DF-fed females may be inferred from the decrease in body mass in this group relative to control DF-fed females (Fig. 3). In contrast, in low dose CADF-fed females, the already low p/c ratio and presence of antibiotic in the CADF diet and loss of microbial functions may have led to the depression of metabolic rates accounting for the lower SMR. This may be similar to what happens during starvation when there is a greater reliance on fat body reserves (Binner, Kloas & Hardewig, 2008; McCue et al., 2015). However, increases in body mass were detected in CADF-fed females relative to control females, suggestive of diet-dependent compensatory feeding by low dose CADF-fed females. Although statistically insignificant, weight gains in low dose and high dose CADF-fed females were relatively higher compared to control CADF-fed females over the seven-day period. The Cohen’s effect size value (*d* = 0.26) between low dose CADF-fed (*N* = 9) (0.07 ± 0.02, g ± S.E.M) and control CADF-fed (*N* = 9) (0.05 ± 0.02) groups suggests a small to moderate practical or biological value of this difference in weight gain, with approximately 58% of the weight gain in low dose CADF-fed females above the mean for the control CADF-fed group (Cohen, 1977). Furthermore, the Cohen’s effect size value (*d* = 0.72) between high dose CADF-fed (*N* = 8) (0.12 ± 0.02) and control CADF-fed (*N* = 9) (0.05 ± 0.02) groups suggests a moderate to high practical or biological value of this difference in weight gain, with approximately 76% of the weight gain in high dose CADF-fed females above the mean for the control CADF-fed group (Cohen, 1977). A possible explanation for the lack of higher SMR in low dose CADF-fed females despite increased body mass (attributed to increased food intake) may be due to an insufficient experimental period. Another explanation may be due to diet-dependent gut bacteria-mediated influences on host SMR responses. Increased food intake and associated increases in body and or gut sizes (Yang & Joern, 1994; Raubenheimer & Simpson, 2003; Behmer, 2008) on the low dose CADF diet may be accompanied by increased intake of the antibiotic, leading to greater reductions in bacterial loads. Thus, lower bacterial loads and loss of microbial nutritional functions coupled with low p/c ratio and antibiotic in CADF diet may have created a situation akin to starvation or food deprivation, leading to depression of metabolic rates and consequently, lower SMR. The relationships between SMR and bacteria load (negatively correlated, *P* = 0.13) and between SMR and change in body mass (positively correlated, *P* = 0.16) in low dose DF-fed females, and the relationships between SMR and bacteria load (positively correlated, *P* = 0.93) and between SMR and change in body mass (positively correlated, *P* = 0.05) in low dose CADF-fed females may be attributed to diet-dependent impacts of lost microbial functions due to reduced bacterial loads.

At the high antibiotic dose, SMR of DF-fed and CADF-fed females decreased to their lowest levels. This is attributed to metabolic suppression in response to very low dietary quality (high antibiotic dose) and loss of microbial functions due to reduced bacterial loads. At this dose, the observed relationships between SMR and bacteria load (negatively correlated, *P* = 0.95) and between SMR and change in body mass (negatively correlated, *P* = 0.75) in high dose DF-fed females, and the observed relationships between SMR and bacteria load (negatively correlated, *P* = 0.92) and between SMR and change in body mass (positively correlated, *P* = 0.05) in high dose CADF-fed females may also be attributed to diet-dependent impacts of lost microbial functions due to reduced bacterial loads. Similar interactions between nutritional contents of diets and gut bacteria on mediating host behavior, food choice, as well as physiology have been observed in *Drosophila melanogaster* (Leitão-Gonçalves et al., 2017). Axenic (no gut bacteria) flies exhibited a compensatory preference for diets enriched with essential amino acids relative to flies with appropriate gut bacteria. Furthermore, essential amino deprivation resulted in longer developmental periods and lowered reproductive outputs in axenic flies relative to non-axenic flies (Leitão-Gonçalves et al., 2017). Results like these provide a context in which the argument can be made that the observed differences in SMR responses between low dose DF-fed and CADF-fed females are mediated by gut bacteria. Although the Chloramphenicol doses used in this study (0.025 and 0.25 mg/ml) were well below reported concentrations (1–5 mg/ml) used in another study (Lin et al., 2015), the impacts on host physiology cannot be definitively excluded. Thus, the use additional antibiotic doses might have been insightful. Furthermore, studies utilizing axenic *P. americana* virgin and non-virgin females generated without antibiotic treatment, in combination with metagenomic and metatranscriptomic approaches, are required to improve mechanistic understanding of the modulating effects of host gut microbial assemblages on host energetic states under different environmental (dietary conditions).

Overall differences in bacterial loads between DF-fed and CADF-fed groups, as well as among control, low and high dose DF-fed and CADF-fed females may be underscored by differences in gut microbiome community composition and function. The primary focus of the current study was the impacts of loss of beneficial microbial functions through disruption of host-gut bacteria metabolic connections on host SMR, and not the impacts on community composition. This is because there are limited and variable effects of dietary shifts on gut microbial community composition in *P. americana* and other related cockroaches. For example, Bertino-Grimaldi et al. (2013) uncovered modest changes in gut microbial community composition in *P. americana* following incubation on two antibiotic-free diets (cellulose and bagasse) for 14 days, as did Perez-Cobas et al. (2015), following incubation of the related cockroach species, *B. germanica* on low and high protein diets for nine days. However, Tinker & Ottesen (2016) uncovered a diverse but comparatively stable and unchanging gut microbiota in *P. americana* following short-term (14 days) and long-term (∼90 days) incubation on eight different diets, one of which was dog food, with greater numbers of individuals. Similarly, Schauer, Thompson & Brune (2014) also uncovered very little changes in gut microbial community composition in a related cockroach species, *Shelfordella lateralis* following incubation of high and low fiber diets for three months. Overall, members of the phyla Bacteroidetes, Firmicutes, and Proteobacteria, were abundant in the gut microbiomes of the cockroaches used in the above-mentioned studies, although some bacterial families are potentially more responsive to dietary shifts than others (Schauer, Thompson & Brune, 2014). Differences in results among these studies may be attributed to the variety of diets used, number of replicates, and sequencing technologies utilized to characterize community composition (Tinker & Ottesen, 2016). Cockroach gut microbial community composition data for control DF-fed and CADF-fed females might have been insightful despite the limited and reported variable effects of dietary shifts on gut microbial community composition in this species. In contrast, dietary shifts or manipulation have a well-documented impact on gut microbial functions in insects. Feeding on high cellulosic diets resulted in reduced gut microbial functions, such as cellulose degradation and provisioning of lactate, acetate, and formate (Kane & Breznak, 1991), and elevated essential amino acid provisioning by gut bacteria (Ayayee, Larsen & Sabree, 2016) in *P. americana*. Furthermore, altering the nutritional composition of artificial diets impacted nutrient provisioning by commensal bacteria in fruit flies (Leitão-Gonçalves et al., 2017), as well as in vertebrates (Krajmalnik-Brown et al., 2012).

In conclusion, we contend that host SMR is mediated in part by the metabolic activity of gut microbial assemblages and that disruption of insect gut microbial assemblages (either through nutrient imbalances or antimicrobials) impacts host SMR. This in turn, can affect fecundity and lifespan (proxies of host fitness) through changes in the allocation of resources to foraging and digestion, rather than energy-intensive processes, such as growth and maintenance (Chown & Gaston, 1999; Chown & Nicolson, 2004). Further studies are required to confirm the roles of gut bacteria in host energetics definitively.

## Supplemental Information

10.7717/peerj.4717/supp-1 Supplemental Information 1 Schema of the experimental design used in this study. Colors represent different experimental groups used.

## Additional Information and Declarations

### Data Availability

The following information was supplied regarding data availability: All data used are presented in Table 1.
