# Supplementary figures and images for: The role of gut microbiota in the regulation of standard metabolic rate in female Periplaneta americana
Source: PeerJ. 2018 May 24;6:e4717. doi: 10.7717/peerj.4717 (PMC5971104; doi:10.7717/peerj.4717)

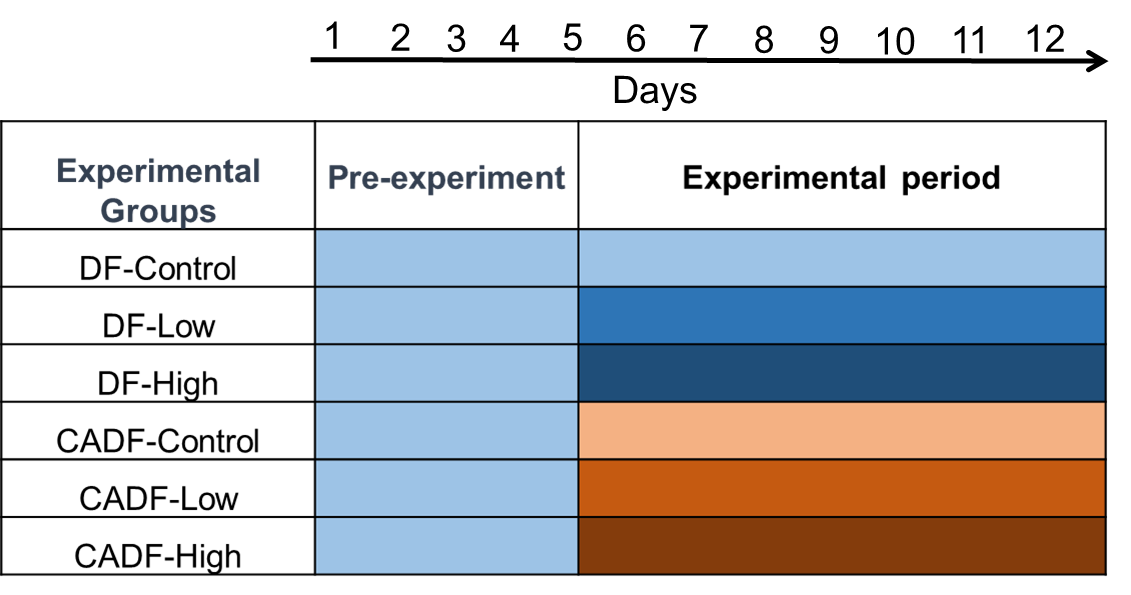

Supplement: Supplemental Information 1 [file peerj-06-4717-s001.png]
